# Dynamic Behaviour of Bridge Girders with Trapezoidal Profiled Webs Subjected to Moving Loads

**DOI:** 10.3390/ma14010038

**Published:** 2020-12-24

**Authors:** Zhiyu Wang, Yunzhong Shi, Xiang You, Ruijuan Jiang, Weiming Gai

**Affiliations:** 1 Key Laboratory of Deep Underground Science and Engineering (Ministry of Education), School of Architecture and Environment, Sichuan University, B1-B2, 10th Floor, Shunji Building, St No. 252, Chengdu 610065, China; 2016223055098@stu.scu.edu.cn; 2 Sichuan Provincial Key Laboratory of Failure Mechanics and Engineering Disaster Prevention & Mitigation, Sichuan University, B1-B2, 10th Floor, Shunji Building, St No. 252, Chengdu 610065, China; 3 Innovation Design Department, Shenzhen Municipal Engineering Design and Research Institute Co., Ltd., 3007 Sungang W Rd, Futian District, Shenzhen 518029, China; jiangrj@szmedi.com.cn (R.J.); gaiwm@szmedi.com.cn (W.G.)

**Keywords:** trapezoidal web, bridge girder, moving load, dynamic analysis

## Abstract

The aim of this study is to find out the degradation of dynamic behaviour of bridge girders with trapezoidal profiled webs when subjected to different vehicle moving loads. Finite element modelling based parametric analysis is demonstrated to be desirable in capturing the dynamic deflection and stress state of critical structural details of girders. The model is validated in the modal analysis through a comparison with theoretical eigenfrequencies. The vibration characteristics are shown to be significantly affected by the corrugation details. The structural service life results of analysed bridge girders are in close agreement with experimental data. It is shown that the dynamic nodal velocity and deflection of analysed bridge girders are greatly affected by the magnitude of the load corresponding to the overload of the vehicle in contrast to the vehicle travel speed. Similar observations can be made for the fatigue life prediction analysis related to the crack initiation when unfavourable effects of the overload vehicle are concerned. Presented analytical results using a fracture mechanics approach could be taken as a good basis for the service life assessment of related bridges with the desired level of performance or functionality.

## 1. Introduction

**Figure 1:**
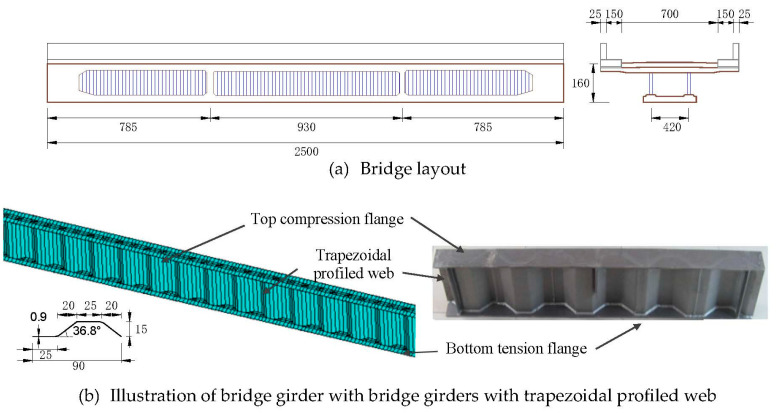
Components of girders with trapezoidal profiled webs (unit for length: cm).

The construction of the plate girders with trapezoidal profiled web in bridge application has been gathering momentum in recent decades. For example, more than 30 such bridges have been constructed during the period from 2005 to 2013 in China. It has been regarded as an effective improvement of shear capacity in comparison with conventional concrete girders and classical composite girders when their corresponding concrete webs and stiffeners have been replaced by the trapezoidal profiled webs. Moreover, since the effectiveness of prestressing has been improved by the accordion effect of the trapezoidal profiled webs, considerable resistance can be achieved especially for prestressed composite girders [1]. The webs are normally designed with the height ranging from 333 to 1500 mm and corrugation amplitude of 40 mm [2]. For weight saving, thin-walled plates with sufficient local and global shear stability as required by design codes are often considered for trapezoidal webs. In the design code of EN 1993-1-5 [3], the supports of web panels are pinned at the edges joining flanges and stiffeners whose torsional stiffness are low. The flange of the concrete slab is beneficial due to the increase in global buckling resistance and extending the linear elastic range of load versus deflection relation. In contrast to conventional prestressed concrete or steel plain web girders, the bridge girders with trapezoidal profiled webs also exhibit a different seismic response due to different torsional and flexural properties of the webs. As outlined in Zhang et al. [4], the seismic waves induced axial forces and bending moment around transverse direction of the trapezoidal profiled web are greater, while the bending moment along the longitudinal direction and the decaying speed of acceleration are less than those of the conventional bridges. Additionally, the combination with stiffer flanges, e.g., concrete filled tubes to increase shear strength [4], and with the use of high strength steel [5] to increase flexural strength, has received more attention by contemporary researchers. Most of those bridges (such as a report in Li et al. [6]) were designed with sufficient shear strength so that the girder resistance is determined by flexural behaviour. An analytical example is a single-span bridge from the support centre lines as shown in Figure 1a. The carriageway is 25 m long and 7 m wide with a 1.5 m footway on either side. The flange steel plates welded at upper and lower end portions of the trapezoidal profiled web are connected to the concrete slab using mechanical shear connectors. For the sake of simplicity in analysis, the steel parts of the bridge girder are isolated consisting of 50 cm wide flange plates and a 160 cm height web with a span of 25 m. The trapezoidal profiled web is 9 mm thick, consisting of a 25 cm wide longitudinal fold and inclined fold, while the wavelength is 90 cm along with web corrugation angle *θ*_c_ = 36.8°, as shown in Figure 1b. The corresponding web height-thickness ratio is 17.78. All configuration and geometric details of the main girder as well as related vehicle moving loads were accounted in the dynamic analysis of this study. The finite element model has been developed using ANSYS 15.0 software (ANSYS Inc. Canonsburg, WA, USA). Please consider this suggested change.

Bridges subjected to repeated loadings during service may suffer problems due to fatigue induced degradation of resistance. Recently, the vulnerability of web-to-flange welded details connecting trapezoidal profiled web and flange plate has been recognized in several fatigue experimental tests of full-size girders and welded joints under flexural bending or tension [7]. The weld toe points connecting the longitudinal fold and the inclined fold of the trapezoidal profiled web are prone to cause cracks on the tension flange. Accordingly, the authors have proposed several methods in the retrofit and improvement of such welded details using CFRP laminates and shot peening onto the tension flanges joining corrugated steel webs [8,9]. Recently, the authors have also investigated the fatigue behaviour of slender webs under in-plane loading in excess of the buckling load. It was found that the fatigue cracks due to predominant shear action propagate much faster than these formed due to combined shear and tearing between the boundary of the tension diagonal and the subpanel of the trapezoidal profiled web [10,11].

Most studies on the fatigue resistance of trapezoidal profiled webs are based on the constant amplitude fatigue loading tests. The analytical results are usually represented based on the nominal stress approach. For example, well-defined nominal stress based *S*–*N* curves are used to give the fatigue resistance of a similar welded detail for the fatigue check known as “FAT Class” in EN1993-1-9 [12] or “Category” in AASHTO [13] and JTG D64-2015 [14]. It is noted that the welded details of the corrugated web to plain flange plates have not been properly addressed in contemporary design code, so the comparison has been made with the detail representing the case of unstiffened plain web to plain flange plate girder. However, these methods based on constant amplitude fatigue test data have certain drawbacks since they fail to reflect the stress developed in the members under dynamic stress states induced by the interaction of the vehicles with the bridges.

The basic understanding of the dynamic behaviour of bridge girders under moving loads is important for the solution of bridge service life and reliability problems, as influenced by the complications of bridge vibration and fatigue. Early field studies on steel railway bridges with different moving loads reported by Fryba [15] demonstrated that the principal bridge girders endure a higher number of stress cycles per year including stress cycles in higher stress-range classes. In contrast, the secondary girders and orthotropic bridge deck suffered lower stress cycles. Deterministic and stochastic approaches have gained good application in the analysis of the response of highway bridges to renewal traffic loads [16]. In theoretical analysis, a Timoshenko beam is generally considered as superior to a Euler-Bernoulli beam for determining the dynamic response of beams. Recently, Sarvestan et al. [17] adopted spectral finite element (SFE) formulation and solution for vibration of cracked Timoshenko beam subjected to moving load. The dynamic stiffness matrix of cracked beam modelled by two massless springs were analysed with both constant velocity and constant acceleration moving load vectors for spectral elements in the frequency-domain. It was found that the remarkable superiority of SFE compared to FE in decreasing the number of elements together with increased numerical accuracy. Zhang et al. [18] performed a 2.5D finite element modelling (i.e., waveguide FE or semi-analytical FE model) and studied the case of the parametric excitation caused by spatial variations in stiffness on a periodically supported beam such as a railway track excited by a moving load. It was found that the Euler-Bernoulli beam model underestimates this parametric excitation due to shear deformation in the rail, which is significant for span lengths less than about 2 m. The results for a moving constant load are not strongly influenced by the vehicle travel speed until the sleeper passing frequency approaches the vertical track resonance and a quasi-static model was concluded as satisfactory for moderate vehicle travel speeds.

For the determination of the general structural performance of bridges in practice influenced by traffic loads, most recent studies focus on the fatigue and fracture evaluation of reinforced concrete bridges using acoustic survey in crack monitoring [19], static ultimate testing [20], finite element analysis [21], corrosion fatigue strength reduction [22], etc. Quantification of traffic load can be made by weight-in-motion (WIM) systems which measure the dynamic axle mass of a moving vehicle to estimate the corresponding static axle mass. WIM has been evaluated in numerous studies including important factors as accuracy, durability, maintainability, ease of installation, portability, and initial and on-going cost as documented in Ref. [23]. Deng and Phares [24] adopted an Automated Ambient Traffic (AAT) approach in the determination of load rating of bridges monitored by a structural health monitoring system under ambient traffic. Its reliability is confirmed in continuously estimating the load carrying capacity of bridges. Shoukry et al. [25] conducted a comparative study between the internal forces in longitudinal girders and deck slabs resulting from traffic loads in the Egyptian codes and proposed modified equations to calculate maximum moments in longitudinal girders. Based on the analytical results, the endurance life of bridges can be further evaluated using the mechanics of fracture and fatigue [26,27]. Additionally, the research methodology into the deformability of structural components in existing buildings related to seismic fragility seismic response has been applied for the bridge components. For example, Ruggieri et al. [26] developed a priori definition about the effective floor deformability of three-dimensional finite element models for which the presence of all elements constituting the entire buildings (floor system, infill panels and elements of retrofit) is accounted for. The seismic fragility is assessed avoiding the possible errors by assuming the floor as rigid. Gentile et al. [26] performed a displacement-based modal or static assessment of continuous bridges with six spans or less. It was shown that this assessment can represent a valid alternative to numerical non-linear static analyses since the resulting performance assessments fell within one standard deviation of the results of the time-history analyses.

Despite above reviewed research work, there is a lack of understanding of the dynamic behaviour of bridge girders with trapezoidal profiled webs subjected to moving loads. In this paper, a finite element modelling based parametric analysis is developed and validated against a simple vibration test in which the nature frequencies and vibration amplitudes are concerned. The resultant modal shapes of the girder with trapezoidal profiled web are then analysed with special attention to the sensitivity of web profiled details to dynamic response. Thereafter, the dynamic deflection and stress conditions of critical structural details of girders are summarized. Based on the modelling results and the fracture mechanics based fatigue life prediction, the dynamic nodal velocity and deflection corresponding to the vehicle travel speed and overload of the vehicle are compared and discussed in comparison with design codes.

## 2. Modelling and Dynamic Analysis

**Figure 2:**
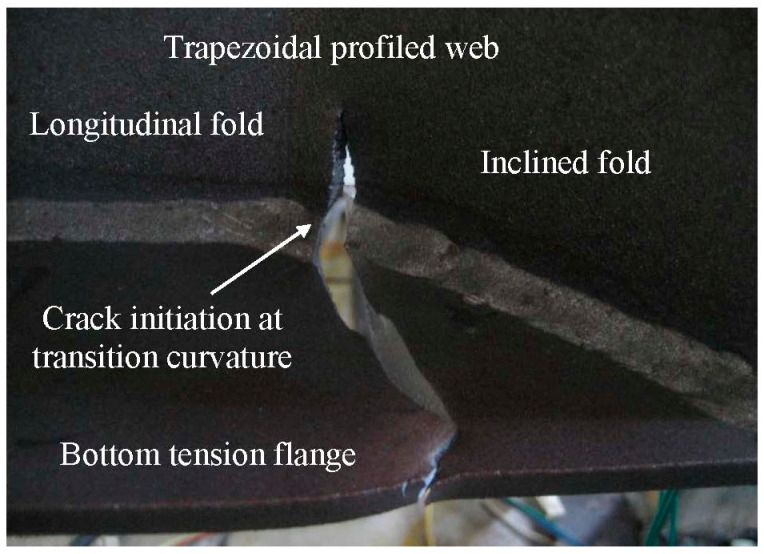
Experimental fatigue crack manner.

**Figure 3:**
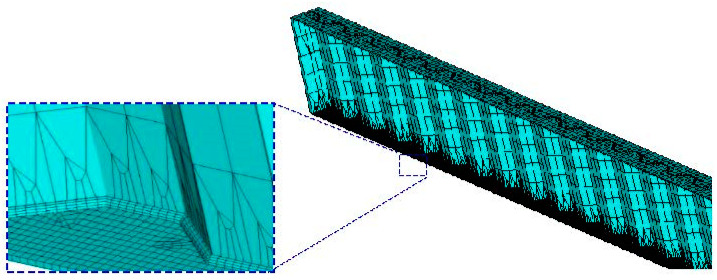
Mesh details of finite element model.

Experimental observation reported in the author’s previous study [7] indicated that fatigue cracks are likely to initiate at the end of the inclined fold intersecting with the transition curvature of the trapezoidal web, as shown in Figure 2. Only the connection detail of the girder is isolated for the concern of fatigue. To further the understanding of dynamic behaviour, a finite element model was developed for the girder with trapezoidal profiled web in same configuration and geometry as shown in Figure 1. The surfaces related to the flanges and the web were merged together that share edges. The element sizes of 2 cm, 5 cm, 15 cm, and 30 cm corresponding 1/45, 1/18, 1/6, and 1/3 of the wavelength, respectively were adopted to study the mesh sensitivity and to calibrate the accuracy of the numerical results. Although the model with 30 mm element size slightly overestimates the stress variation at the transition curvature of the corrugation, using the mesh sizes of 2 cm and 5 cm give a similar and very good estimation of stress distribution as calibrated against the test results reported in Ref. [7]. Accordingly, fine meshes (approximate element size of 5 cm) are adopted for the adjacent regions of the welded connection between the web and the flange on the tension flange while relatively coarse meshes are adopted for the rest regions as shown in Figure 3. The element used for the modelling was chosen from the ANSYS element library as Shell 181 elements, which have four nodes with six degrees of freedom at each node. The steel material models are defined in the modelling according to the test data as listed in Table 1; the elastic modulus and the Poisson’s ratio are taken as *E*_s_ = 206,000 MPa and *υ* = 0.3 respectively.

**Figure 4:**
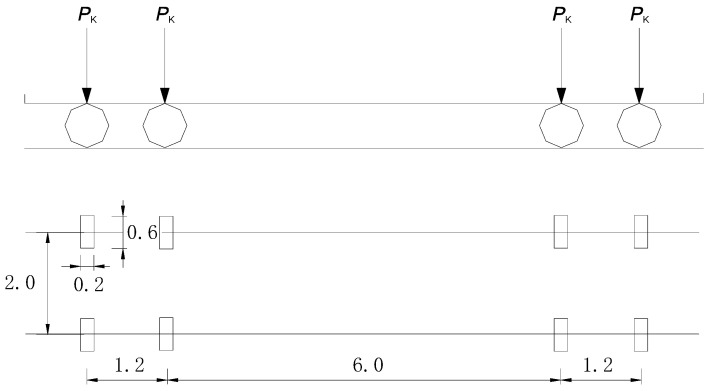
Diagram of moving load model (unit for length: m) [14].

The dynamic load is simulated using the fatigue load model suggested by JTG D64 [14] which is widely adopted for the fatigue computation of structural members in contact with wheels. Following the design guidance, the standard vehicle load, as schematically plotted in Figure 4, is applied onto the girder successively. According to the position of the wheel entering the bridge, the number of nodal forces is increased with the increment of *P*_k_ to 4 *P*_k_ (i.e., the nodal assembly of four forces applied along the longitudinal direction of the girder is shown in Figure 3). Conversely, as the vehicle leaving the bridge, the number of nodal forces is decreased with the decrement of 4 *P*_k_ to 0. The time for moving load to arrive *i*th node is *x*_i_/*v*, where *x*_i_ is the node location. The maximum and minimum stresses caused by the dynamic loading are then compared against the allowable fatigue stresses. Five loading cases are considered for the modelling the dynamic behaviour of the girders under different fatigue load travelling velocity and increased vehicle load as listed in Table 2. The edge of the top flange is laterally restrained to exclude torsion and out-of-plane deflection. For simplicity, the bridge damping, road roughness, and the interaction between the vehicle and bridge are ignored and the dynamic moving vehicle load is directly applied on the nodes of the top flange of the girder. According to the position of the moving load, three phases of nodal force assembly are considered in proper sequence as: front wheel load only, front wheel load + rear wheel load (as examplified in Figure 3), and rear wheel load only. Assuming the location of *i*th node from the support is *x*_i_ and the moving velocity is *v*, the time for the load to arrive *i*th node is *t*_i_ = *x*_i_/*v*. Using ANSYS parameter design language (APDL), a program for dynamic modelling is compiled in the following steps: (1) developing finite model with proper mesh discretization adapting to the moving load location; (2) checking whether the instant load location of the front wheel and the rear wheel are within the span of the girder or not; (3) reading the proper loading case and apply the nodal forces on the girder model using transient analysis in ANSYS.

For the sake of ensuring their reliability, and especially their stability and serviceability, it is important to analyse the bridge structure loaded by dynamic excitation. Analytical modal Analysis is the process of characterizing the dynamic response of a system in terms of its modes of vibration. The eigenfrequency for the girders with the trapezoidal profiled webs are varied with the geometric features of the web whose shear modulus can be introduced from the expression given by Johnson and Cafolla [28] as

(1) $$G_{w}=\frac{E(b_{l}+b_{i}\cos\theta_{c})}{2(1+\upsilon )(b_{l}+b_{i})}$$

where, *b*_l_ and *b*_i_ are the lengths of longitudinal fold and inclined fold respectively. *θ*_c_ is the corrugation angle.

Accounting the longitudinal displacement difference function and section angular displacement function, the deflection function can be obtained based on the suggested formula in [29], and then the eigenfrequency for the bending of girders with trapezoidal profiled webs can be written as

(2) $$\omega_{c}=\gamma_{0}\omega_{n}=\gamma_{0}{\left(\frac{n\pi }{l_{0}^{}}\right)}^{2}{\left(\frac{EI}{m}\right)}^{0.5}$$

where, *γ*_0_ is the correction coefficient converted from original form for the cross section of box girder as

(3) $$\gamma_{0}={\left[1+\frac{5Eb_{0}^{2}}{112G_{m}}{\left(\frac{n\pi }{l_{0}}\right)}^{2}\right]}^{0.5}{\left[1+\frac{EI}{G_{w}A_{w}}{\left(\frac{n\pi }{l_{0}}\right)}^{2}+\frac{5Eb_{0}^{2}}{14G_{m}}{\left(\frac{n\pi }{l_{0}}\right)}^{2}+\frac{5E^{2}b_{0}^{2}I}{112G_{w}G_{m}A_{w}}{\left(\frac{n\pi }{l_{0}}\right)}^{4}\right]}^{-0.5}$$

where, *G*_m_ and *b*_0_ are the shear modulus and breadth of the flange respectively. *A*_w_ is the cross section area of the web and *l*_0_ is the span of the beam.

## 3. Results and Discussion

### 3.1. Numerical Verification

**Figure 5:**
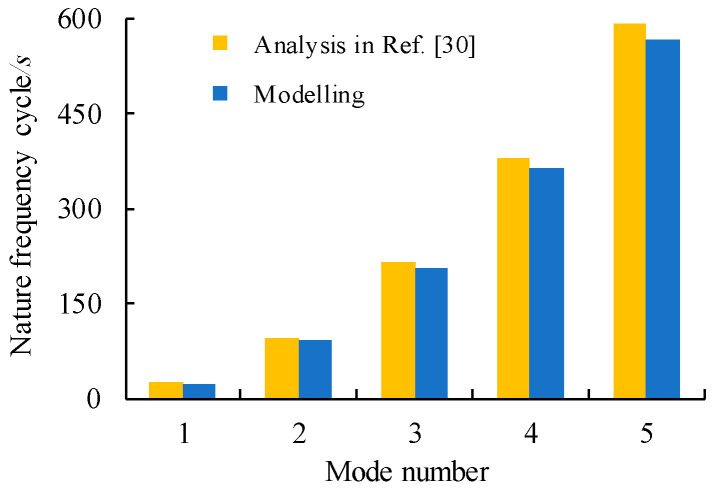
Comparison of nature frequencies.

**Figure 6:**
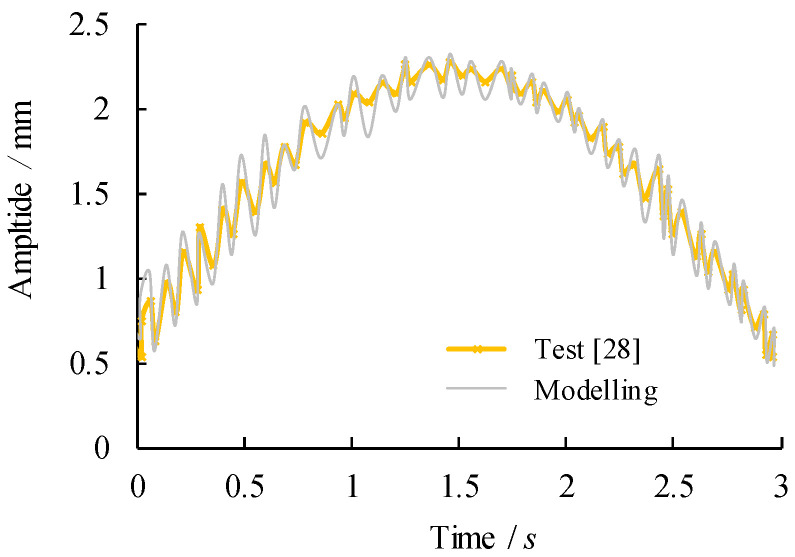
Comparison of dynamic amplitude of mid-span.

Unlike full-scale bridge structures where the dynamic properties of constituent components are inherently varied with the construction deficiencies and adverse environmental factors, a relatively small-scale beam specimen is desired for the sake of finite element model calibration since the errors caused by these effects may be somehow excluded. For the above-mentioned reason, modelling of a simply support small-scale beam excited under moving load reported in [30] allows verifying the correlation of modelling and experimental natural frequencies and dynamic amplitude. The moving load travelled at uniform speed over the beam span from the starting point of 7 cm to the end point of 72 cm from global coordinates centre at end support. The moving load is applied by implementation of a carriage with negligible sliding in contact with the beam and minimum attached area. The parameters of the moving loads are taken as the load magnitude of 39.24 N and uniform load travel speed of 0.2 m/s. The beam was simply supported at the ends with rolling support on one side end and pinned at the other side. In the reported test in Ref. [30], the nature frequency was measured in cycles per second with units of Hertz and the dynamic acceleration of a physical device as a voltage was measured using an accelerometer. The resultant natural frequencies and vibration of the simply supported beam are plotted in Figure 5 and Figure 6 respectively. The natural frequencies were also found by analyses which were done by finite element analysis software ANSYS 15.0 software (ANSYS Inc. Canonsburg, WA, USA). Through adding instantaneous excitation along the beam, the model has been solved using embedding algorithm of governing equation related to the mode shapes and eigenfrequencies. The modelling natural frequency and mode amplitudes of the model are also summarised in Figure 5 and Figure 6. The adequacy of the developed model is verified against test data and their related curve as the evidence of a good agreement in plotted comparison.

### 3.2. Modal Analysis

**Figure 7:**
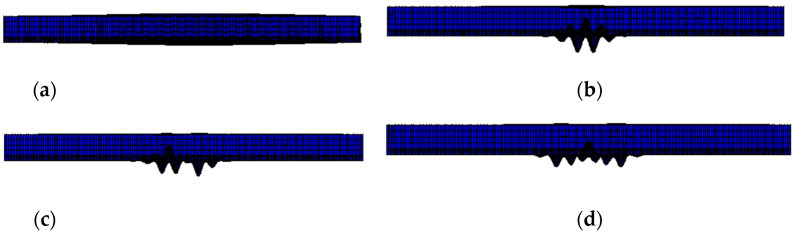
Summary of typical four mode shapes of vibration. (**a**) 1st mode shape of vibration. (**b**) 2nd mode shape of vibration. (**c**) 3rd mode shape of vibration. (**d**) 4th mode shape of vibration.

According to the dynamic analytical model of the girders with trapezoidal webs established, the structural natural vibration characteristics are investigated. The typical vibration mode shapes are shown in Figure 7. The first mode shape for the girder with trapezoidal profiled web is an in-plane pure bending mode along the g the axis of the member only with notable symmetrically distributed deflection along the midspan of the girder. The nature frequency for the first order natural mode shape of vibration is 20.78 Hz which is very close to 19.17 Hz as calculated from Equation (2). The second mode shape of vibration is very local deflection at the bottom tension flange in the vicinity of the midspan of the girder while rest parts of the girder almost remain unchanged. The third mode shape of vibration exhibits a series of crumpling at the midspan of the bottom tension flange and its region between adjacent inclined folds deflects significantly. Similar observation can be made for the fourth mode shape of vibration which is featured by some more defection shapes at the bottom tension flange. This indicates the corrugation details at the midspan near the bottom tension flange is very sensitive to vibration.

### 3.3. Dynamic Analytical Results Concerning Varied Vehicle Velocity

**Figure 8:**
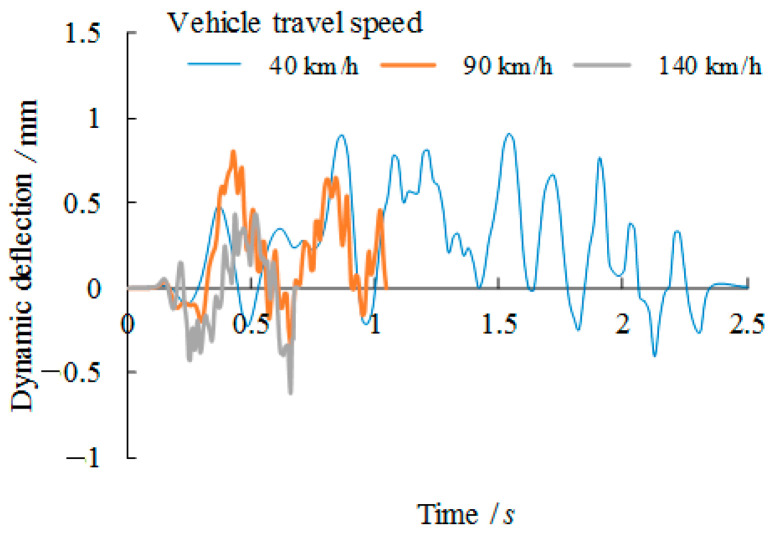
Comparison of dynamic deflection versus vehicle travel speed relation.

**Figure 9:**
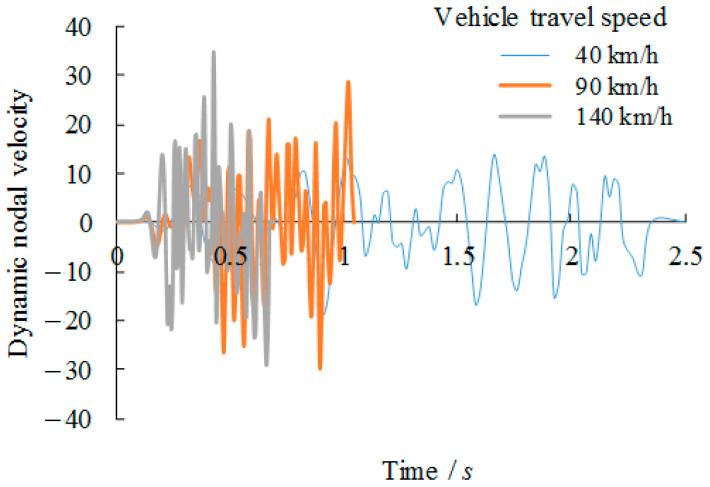
Comparison of nodal dynamic velocity versus vehicle travel speed relation.

The dynamic behaviour of the vehicle passing the bridge girders with trapezoidal profiled webs is firstly compared with varied travel speeds of 40 km/h, 90 km/h and 140 km/h. Related dynamic deflections and nodal velocities of the girder at the midspan are compared in Figure 8 and Figure 9 respectively. As plotted in these figures, although these curves are very close at the very beginning of loading, the increase in the vehicle travel speed to 90 km/h and 140 km/h contributes to 25% and 51% increase of the vertical negative deflection respectively against the deflection related to the vehicle travel speed of 40 km/h. In contrast, more significant increases, i.e., 208% and 252% respectively, are observed for the nodal velocities at the midspan location. This suggests that the dynamic behaviour is notably amplified due to the increase of the vehicle travel speed.

**Figure 10:**
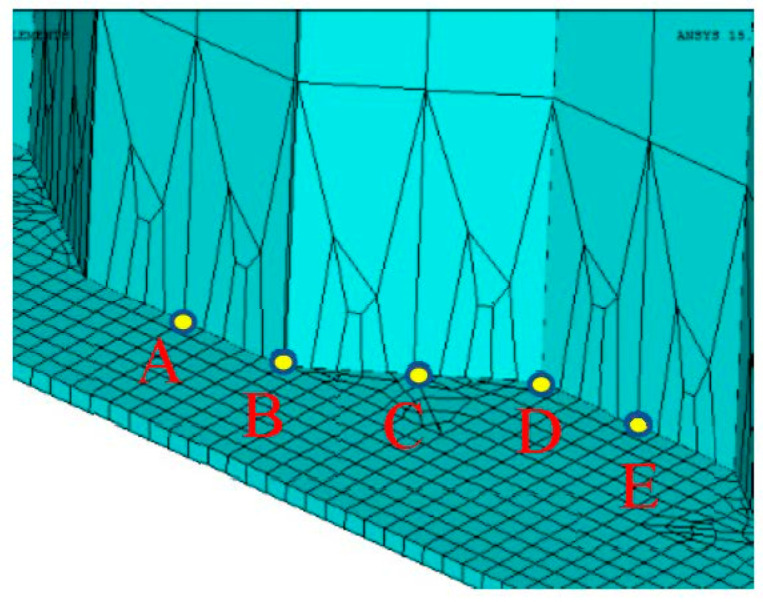
Corrugation characteristic points.

**Figure 11:**
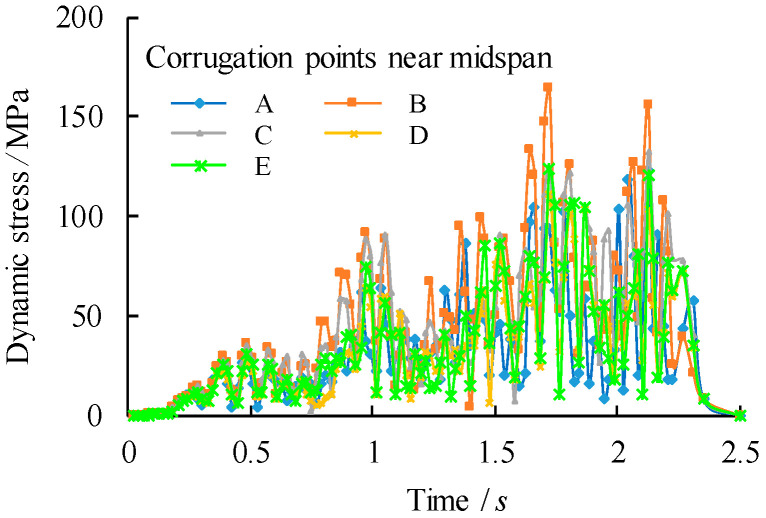
Comparison of dynamic stress distribution of the corrugation near midspan centreline (*v* = 40 km/h).

**Figure 12:**
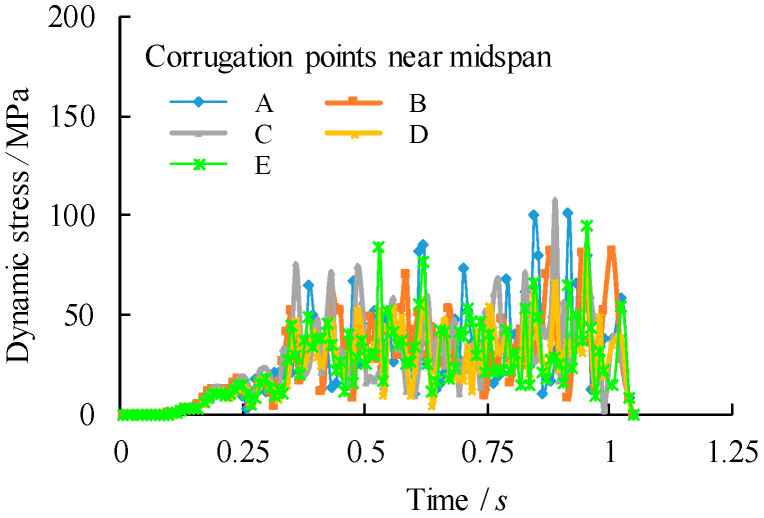
Comparison of dynamic stress distribution of the corrugation near midspan centreline (*v* = 90 km/h).

**Figure 13:**
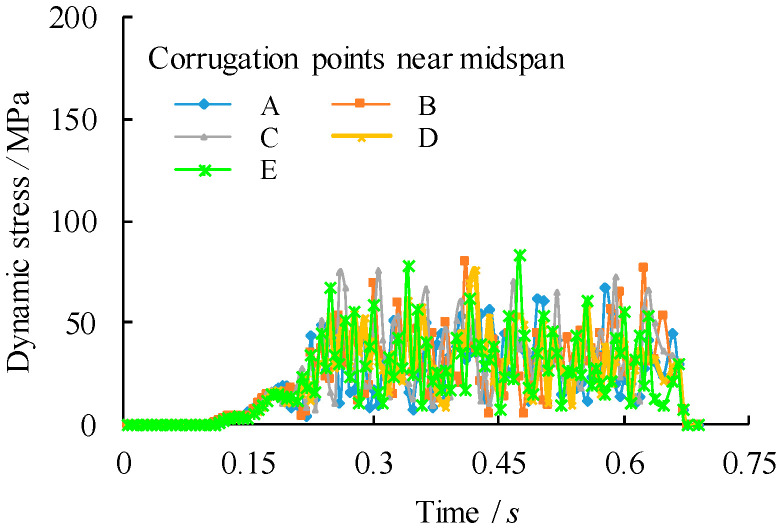
Comparison of dynamic stress distribution of the corrugation near midspan centreline (*v* = 140 km/h).

**Figure 14:**
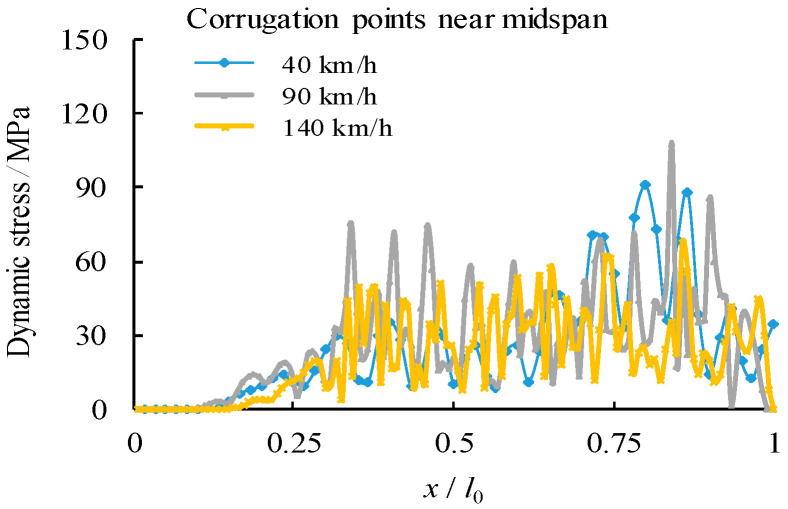
Comparison of dynamic stress of point B varied with vehicle travel speed.

To compare the stress states at the midspan, five corrugation characteristic points are chosen within a half wave length away from the midspan centreline as from point A to point E, in which the points B and D are intersection points, the points A and E are middle points of the longitudinal fold and the point C is the middle point of the inclined fold, as shown in Figure 10. Their corresponding von Mises stress distributions are shown in Figure 11, Figure 12 and Figure 13. It can be observed that the dynamic stress is low as the front wheel load is applied on the girder and subsequently increased until the rear wheel load approaching the end of the support. The magnitude of the stresses at point B is as high as equivalent to these at the midspan of the girder although there is a location difference with a quarter wave length of the trapezoidal web. For the vehicle travel speed of *v* = 40 km/h, there is a notable variation of the stress distribution with the maximum stress at 1.72 s. In contrast, such a variation of peak stress becomes gradual as the vehicle travel speeds are increased to *v* = 90 km/h and 140 km/h. As a further examination, the stress distribution of the point B is plotted against the normalized moving position away from the origin with respect to the span of the girder (*x*/*l*_0_), as shown in Figure 14. It is evident that the increase in vehicle travel speed has a limited effect on the increase in peak stress. This can be explained as the load passing through the girder in a very short time so that the local connecting part of the structure is less stressed.

### 3.4. Dynamic Analytical Results Concerning Overload Vehicle Condition

**Figure 15:**
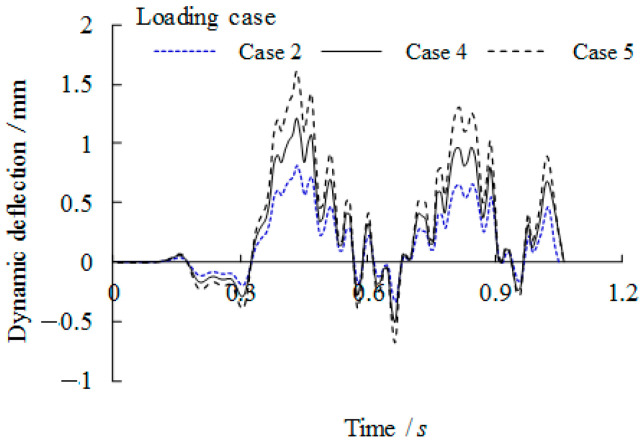
Comparison of dynamic deflection with varied overload cases (Case referring to Table 2).

**Figure 16:**
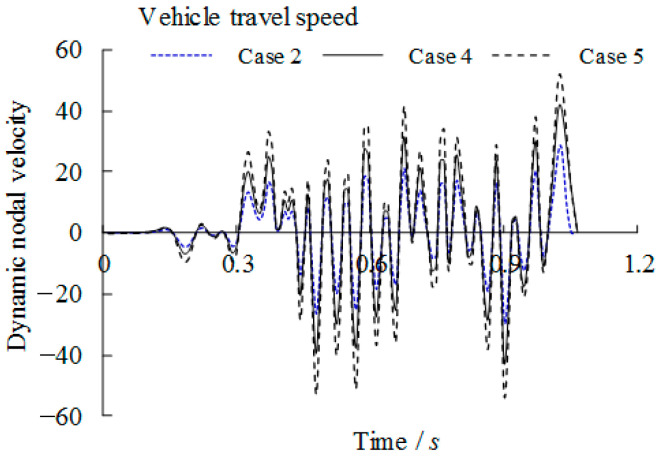
Comparison of nodal dynamic velocity with varied overload cases (Case referring to Table 2).

**Figure 17:**
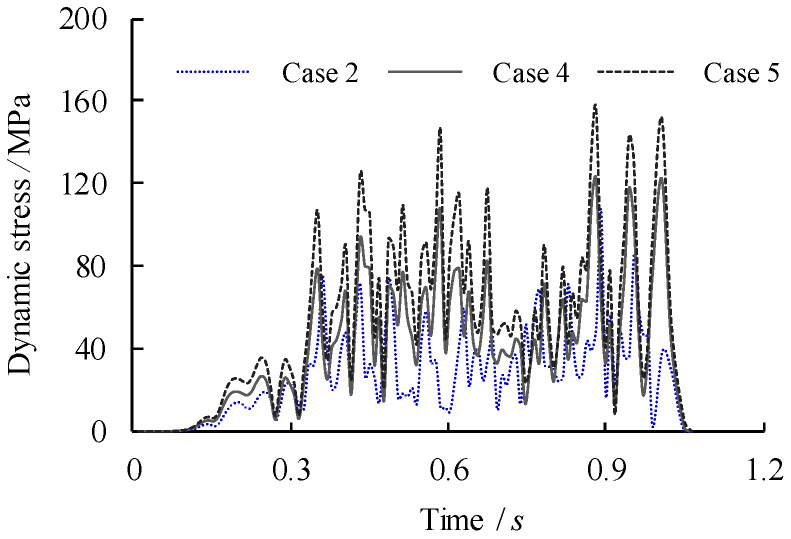
Comparison of dynamic stress of point B with varied overload cases (Case referring to Table 2).

To study the effect of overload of the vehicle on the dynamic behaviour of the bridge girders with trapezoidal profiled webs, the vehicle passes the bridge girders with an initial travel speed of *v* = 90 km/h. Related dynamic deflections and nodal velocities of the girder at the midspan are compared in Figure 15 and Figure 16 respectively for which the loading case can be referred to Table 2. It can be seen that the both the peak deflection and the nodal velocity are increased linearly as the vehicle load is increased to 1.5*P*_k_ and 2*P*_k_. Given high stresses at the intersection of the longitudinal fold and the inclined fold of the trapezoidal web as mentioned previously, the dynamic stresses of point B under three loading cases are compared in Figure 17. Much greater amplification of dynamic peak stresses is observed with the increase of the applied vehicle load in contrast to the increase of the vehicle travel speed. This suggests that the critical dynamic behaviour of the bridge girders with trapezoidal profiled webs is more sensitive to the overload vehicle in contrast to overspeed vehicle.

## 4. Fatigue Life Prediction Based Fracture Mechanics Method

Following the theory of fracture mechanics and the Paris law [25,26,31], the fatigue life of welded details can be obtained by assuming the initial crack is propagated through the flange plate, as follows:

(4) $$N=\frac{1}{C}\int_{a_{0}}^{t_{f}}\frac{1}{\Delta K^{m}}da$$

where, *t*_f_ and *a*_0_ are the steel flange plate thickness and initial crack depth as 0.12 mm [25]. *C* and *m* are the material constants which can be taken as 1.3 × 10^−12^ and 3 respectively [32]. The stress intensity factor, Δ*K*, can be defined by assuming that a semi-elliptical crack propagation in depth at the weld toes as

(5) $$\Delta K=F_{S}F_{E}F_{T}F_{G}\Delta \sigma \sqrt{\pi a}$$

where *F_S_* is a free surface correction factor, *F_E_* is a crack shape correction factor, *F_T_* is a finite thickness correction factor, *F_G_* is a geometry correction factor, Δ*σ* is an applied stress range. The ratio of the crack depth, *a*, to the crack length, *c*, is assumed as equal to 0.75. *θ* is the integration parameter. Each correction factor can be obtained from the following expressions:

(6) $$F_{S}=1.12-\frac{0.12a}{c}=1.03$$

(7) $$F_{E}=\frac{1}{E(k)}$$

(8) $$E(k)=\int_{0}^{\frac{\pi }{2}}{\left[1-\left(1-\frac{a^{2}}{c^{2}}\right)\sin^{2}\theta \right]}^{0.5}d\theta =\int_{0}^{\frac{\pi }{2}}{\left[1-0.44\sin^{2}\theta \right]}^{0.5}d\theta$$

(9) $$F_{T}={\left[\sec\left(\frac{\pi a}{2t_{f}}\right)\right]}^{0.5}$$

(10) $$F_{G}=\frac{K_{t}}{1+0.88a^{0.576}}$$

(11) $$F_{G}=\frac{K_{t}}{1+0.88a^{0.576}}$$

**Figure 18:**
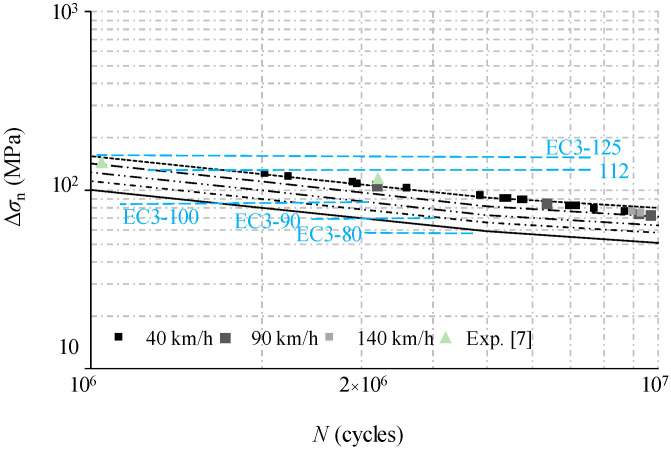
Comparison of experimental and predicted fatigue life varied with vehicle travel speed.

**Figure 19:**
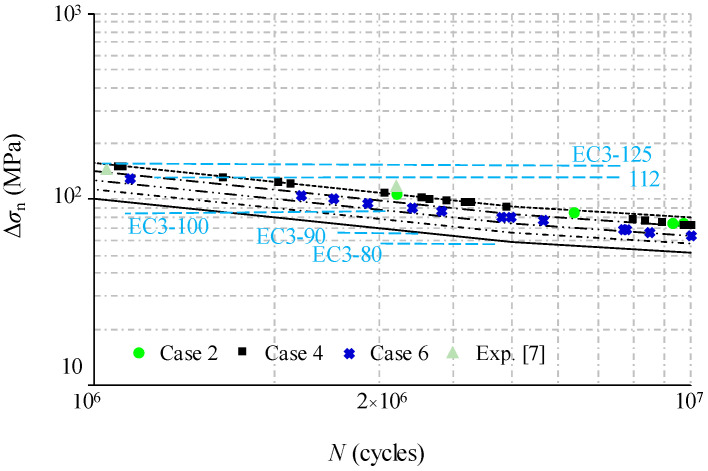
Comparison of experimental and predicted fatigue life with varied overload cases.

Substituting the dynamic stress results from finite element modelling as Δ*σ* in Equation (10), the fatigue life can be predicted by substituting Equation (10) into Equation (9) and using logarithmic transformation. When plotted on log–log scales, the relationship between alternating dynamic stress and the predicted fatigue life can be described in Figure 18 and Figure 19. Based on the fatigue test results, a constant value of *m* = 3 is used for a comparison with the fatigue detail categories in the design code of EN 1993-1-9 [32]. In this code, the specified details are given not for trapezoidal profiled webs but for girders with flat webs in detail categories of 112 or 125, and for girders with welded transverse stiffeners in the detail category of 80. Additionally, the fatigue experiment results from reference [8] are plotted together with analytical fatigue life versus stress range on log–log scales. It can be observed that the general trend of prediction data is very close to the experimental data of the standard loading case. The fatigue life data are very close and stable, approaching the detail category of 100 as the vehicle travel speed is increased from 90 km/h to 140 km/h. In practice, this justifies the restriction of the vehicle travel speed to no more than 80 km/h, as required by the road administration for the exemplary bridge case. On the other hand, the predicted fatigue life for the specimen under loading case 4, when compared to the loading case 2, is decreased slightly to the detail category 110. In contrast, such a decrease for loading case 6 is greatly amplified to the detail category of 90 which is just slightly higher than the detail category for welded transverse stiffeners. In this context, the girder suffers from more than twice the vehicle weight limits of a four-axle truck (i.e., 31 tons gross vehicle weight) required by the road administration for the exemplary bridge case. This indicates that the fatigue life of the bridge girders with trapezoidal profiled webs is more likely to decrease along with the stress concentration induced by the overload vehicle. Therefore, the limitation of the maximum required load of the vehicle is recommended for the maintenance of such bridge girders with trapezoidal profile webs. It is noted that above results of the analysis related to the crack initiation are based on a fracture mechanics approach, so further study is required for the service life assessment of the bridge when the desired level of performance or functionality is concerned.

## 5. Concluding Remarks

The dynamic behaviour of bridge girders with trapezoidal profiled webs subjected to moving loads has been introduced in this paper. Theoretical basis related to eigenfrequency has been reviewed and compared to the analytical results for the girder with the trapezoidal profiled web. The proposed finite element model has been validated against the referred test data in terms of the natural frequencies and the vibration amplitudes. The modal characteristics have been examined and the effects of the vehicle travel speed and overload on the structural dynamic behaviour have been analysed in detail. Based on the fracture mechanics approach, the fatigue life analysis has been conducted for crack propagation of the critical welded details of the girder in depth. The analytical results have been compared with codified detail categories related to varied loading cases of the vehicle travel speed and weight. The following conclusions can be drawn:

- The results of the developed finite element model incorporating proper boundary condition and corrugation details agree well with referred excitation test and theoretical results in terms of natural frequencies and vibration amplitudes. Apart from the deflection induced by the bending moment, the trapezoidal profiled web connecting part at the midspan of the bottom tension flange is sensitive to vibration in the form of localized deflection and crumpling.
- The dynamic deflection and nodal velocity are notably increased with the increase in vehicle travel speed. The dynamic stress is low as the front wheel load is applied on the girder and subsequently increased until the rear wheel load approaching the end of the support. Due to very short time of load passing, the peak stress at the tension flange is marginally affected by the variation of the vehicle travel speed. The resultant stresses are relatively high at the intersection of the trapezoidal profiled web and located at a quarter wavelength of the trapezoidal web away from the midspan of the girder.
- The overload vehicle has adverse effect on the girder dynamic behaviour subjected to moving loads since its resultant dynamic stress at the intersection of the longitudinal fold and the inclined fold of the trapezoidal web is almost linearly amplified. Moreover, the resultant fatigue life is greatly reduced in contrast to that caused by the effect of the vehicle travel speed from above the detail category 100 to no more than the detail category 90; thus the limitation of the maximum required load of the vehicle is required when the maintenance of such bridge girders is concerned. Additionally, above understanding could be taken as a basis for the assessment of the service life of the bridge providing the desired level of performance or functionality with any required level of repair and maintenance as a following up study.

## Figures and Tables

**Table 1:** Mechanical properties of test materials.

Component	Chemical Composition (%)					Mechanical Properties		
	C	Si	Mn	P	S	Yield Stress [MPa]	Ultimate Stress [MPa]	Elongation/%
Flange	0.16	0.33	1.36	0.035	0.022	480	565	25
Web	0.14	0.32	0.48	0.045	0.04	410	495	22

**Table 2:** List of loading cases.

Load Case	*P*_k_ [kN]	*v* [km·h^−1^]	Passage Time [s]	Overload
1	120	40	2.25	×
2	120	90	1.00	×
3	120	140	0.64	×
4	180	90	1.00	√
5	240	90	1.00	√

## Data Availability

Data sharing is not applicable to this article.
